# Valuable Remedy for Headache

**Published:** 1884-12

**Authors:** 


### A VALUABLE REMEDY FOR HEADACHE.

WE desire to call attention to a simple, and at the same time wonderfully efficient, treatment for many kinds of headache. We lay no claim to originality, nor do we know who the originator was, but having used it for a year or more, and in many cases with remarkable results, we feel disposed to give it our endorsement, and desire to make it more generally known. The remedy is nothing more nor less than a solution of the bi-sulphide of carbon. A with-mouth glass stoppered bottle is half filled with cotton or fine sponge, and upon this two or three drachms of the solution are poured. When occasion for its use occurs the mouth of the bottle is to be applied to the temple or as near as possible to the seat of pain, so closely that none of the volatile vapor may escape, and retained there four or five minutes or longer. For a minute or so nothing is felt, then comes a sense of tingling, which in a few minutes—three or four usually—becomes rather severe, but which subsides almost immediately if the bottle be removed, and any redness of the skin that may occur will also quickly subside. It may be reapplied if necessary, several times in the day, and it generally acts like magic, giving immediate relief.

We believe this was the basis of a once popular nostrum. The class of headaches to which it seems especially adapted is that which may be grouped under the broad term of "nervous." Thus neuralgic, periodic and hysterical headaches, and even many kinds of dyspeptic headaches, are almost invariably relieved by it. True the relief of a mere symptom is quite another thing from the removal of the cause, yet no one who has had the distress and even agony caused by severe and frequent recurring headaches (and who has not seen it?) but will rejoice to be able to afford relief in so prompt and simple a manner, besides it is sure to secure the hearty gratitude of the patient if he has suffered long. As to the modus operandi we have nothing more definite than a theory to offer, and that is that the vapor being absorbed through the skin produces a sedative effect upon the superficial nerves of the parts to which it is applied. We know by experiment that its influence is not due to its power as a counter-irritant. We, however, know that it does act, and if we do not clearly see in what way it acts that is no more than can be said of several other remedies which are firmly established in professional favor and confidence.—*Physicians' and Surgeons' Investigator.*

---

To educate a youth so that he shall have a strong moral character, do not isolate him, but teach him to come out unscathed from temptation.

